# Biliary Complications After Surgery for Hydatid Disease: A Five-Year Experience in a Tertiary Care Center

**DOI:** 10.3390/healthcare13091077

**Published:** 2025-05-06

**Authors:** Sebastian Vâlcea, Bogdan Cristian Dumitriu, Mircea Beuran, Catalin Vladut Ionut Feier

**Affiliations:** 1 Department of Surgery, Carol Davila University of Medicine and Pharmacy, 8 Eroii Sanitari, Sector 5, 050474 Bucharest, Romania; sebastian.valcea@umfcd.ro (S.V.); mircea.beuran@umfcd.ro (M.B.); 2 Department of Surgery, Clinical Emergency Hospital Bucharest, 014461 Bucharest, Romania; 3 Abdominal Surgery and Phlebology Research Center, “Victor Babes” University of Medicine and Pharmacy, 300041 Timisoara, Romania; catalin.feier@umft.ro; 4 First Surgery Clinic, “Pius Brinzeu” Clinical Emergency Hospital, 300723 Timisoara, Romania

**Keywords:** surgical outcomes, biliary fistula, postoperative complications, cystic echinococcosis, hepatic hydatid cyst

## Abstract

Background and Objectives: Cystic echinococcosis (CE) remains a significant health concern in endemic areas, including Romania, where hepatic hydatid cysts frequently require surgical treatment. Surgery represents the cornerstone of therapy, particularly in large, complicated, or symptomatic cysts, where medical or minimally invasive options may be insufficient. This study aims to investigate the clinical characteristics, risk factors, and postoperative evolution of patients undergoing surgical intervention for hepatic CE in a tertiary care center over a five-year period. Materials and Methods: This retrospective study examined data from 62 patients who underwent surgical procedures for hepatic CE during a 5-year period. The analysis focused on demographic parameters, cyst morphology, surgical techniques employed, and postoperative complications, with particular attention to the frequency, management, and outcomes of biliary fistulas. Results: The study cohort had an average age of 44.1 years, with a slight predominance of female patients (51.6%). The majority of cysts (62.9%) were located in the right hepatic lobe, with an average diameter of 10.9 cm. Postoperative complications were recorded in 25.8% of cases, with biliary fistulas being the most frequent (12.9%). Patients who developed biliary fistulas presented significantly larger cysts (152.13 ± 105.68 mm vs. 102.20 ± 37.86 mm, *p* = 0.012) and required an extended length of hospitalization, particularly in high-output cases (29 vs. 9.3 days, *p* = 0.045). Hospital stays and treatment expenses were notably higher among patients with biliary fistulas. Conclusions: Biliary fistulas were observed exclusively in patients who underwent partial cystectomy. This finding highlights the need for increased caution when performing partial cystectomy, especially in cases involving large or recurrent cysts, where the risk of postoperative biliary fistulas is higher. Tailoring the surgical technique based on cyst characteristics and incorporating intraoperative strategies to manage or prevent biliary leakage may help reduce morbidity. Early identification and multidisciplinary management of high-risk cases are key to improving outcomes in hepatic CE.

## 1. Introduction

Cystic echinococcosis (CE), also known as hydatid disease, is a zoonotic infection of major global public health significance caused by the larval stage of the Echinococcus granulosus parasite [1]. This parasitic disease remains endemic in numerous regions worldwide, including Eastern Europe, Central Asia, North Africa, and Latin America, exerting a substantial burden on healthcare systems and local economies [2]. Human infection tends to peak during the fourth and fifth decades of life. It occurs through the ingestion of eggs excreted by definitive hosts (dogs and other canids), which, once ingested, lead to the development of hydatid cysts predominantly in the liver and lungs. However, other organs may also be affected [3]. Romania’s rural population constitutes about 45% of its total populace, with a significant proportion involved in agricultural activities on a daily basis [4]. Notably, Romania has been identified by the World Health Organization as an endemic region for specific health issues [2]. This designation is substantiated by elevated incidence rates observed across several counties between 1991 and 2008, which varied between approximately three to nine cases per hundred thousand residents [5].

Upon the ingestion of Echinococcus granulosus eggs in the human host environment, these release oncospheres are capable of penetrating through the intestinal wall and disseminating hematogenous material throughout the body. This process leads to the formation of hydatid cysts predominantly within hepatic tissues (60–70% of cases), followed by pulmonary involvement (20–25%). Less common sites include splenic renal cerebral or osseous regions where such cysts may also develop, albeit more rarely [6,7]. The evolution of these cysts is characterized by slow growth and an asymptomatic initial phase during which they do not typically cause noticeable symptoms until they have expanded sufficiently to compress adjacent structures [8,9]. Clinical manifestations are generally nonspecific but may include symptoms such as abdominal pain, hepatomegaly, and obstructive jaundice—particularly when the cysts are located in the liver—or dyspnea if the cysts are situated within pulmonary tissues [10,11,12]. In the absence of appropriate treatment, cystic echinococcosis can progress towards severe complications, including rupture with subsequent peritoneal dissemination secondary infections and biliary fistulas, among other serious health issues that may arise from untreated disease progression [13].

The management of cystic echinococcosis is contingent upon the size, location, and characteristics of the lesions, following current international recommendations, particularly those provided by the WHO Informal Working Group on Echinococcosis (WHO-IWGE) [14]. Initial assessment includes imaging techniques—such as ultrasound and CT—to establish the type and stage of the hepatic cystic echinococcosis, which are crucial in determining the appropriate therapeutic pathway. Surgical treatment remains the therapeutic standard for voluminous, complicated, or symptomatic lesions; however, it is associated with postoperative risks, including biliary fistulas and recurrences [8,15]. Over the past two decades, minimally invasive techniques have emerged as effective alternatives in selected cases. Minimally invasive techniques include percutaneous imaging-guided drainage (PAIR—puncture, aspiration, injection, re-aspiration), which has significantly reduced postoperative morbidity [13,16]. The injection phase involves introducing a scolicidal solution into the cyst. In most cases, agents such as hypertonic saline (15–20%) or absolute ethanol are used, both being effective in neutralizing viable parasitic elements and minimizing the risk of recurrence or dissemination. This method is indicated for small to medium-sized cysts without communication with the biliary tree. PAIR represents an efficient procedure allowing strict control over cyst content and preventing parasitic dissemination during medical interventions [16]. Albendazole therapy is recommended both before and after surgical interventions or PAIR procedures. It should be administered at least one month prior to open surgery and for two months following the procedure. Albendazole plays a crucial role in preventing parasitic recurrences by inactivating protoscolexes within hydatid cysts [7,17].

Despite advancements in surgical techniques and the integration of chemotherapy, recurrence remains a significant concern in the treatment of hydatid disease. Recurrence rates for CE vary widely, ranging from 0% to 22%, and can occur at intervals spanning from three months to twenty years following initial surgery. Recurrences are often linked to severe complications such as intrabiliary rupture, anaphylactic reactions, and pyogenic infections [18]. These complications underscore the need for vigilant postoperative monitoring and comprehensive management strategies.

This study aims to analyze the clinical presentation of human cystic echinococcosis and the postoperative outcomes in patients hospitalized in the surgical department of a tertiary care center in Romania over a five-year period. In order to avoid the confounding effects introduced by major systemic disruptions, the study focused exclusively on the pre-pandemic period. The COVID-19 pandemic, which officially reached Romania on February 26, 2020, has been associated with significant shifts in healthcare accessibility and a documented decline in surgical interventions across the country [19,20,21,22,23,24,25]. Cystic echinococcosis was no exception, with pandemic-related disruptions affecting both diagnosis and treatment, as reported in both global and Romanian contexts [26,27]. Therefore, limiting the analysis to the pre-pandemic interval allows for a more accurate reflection of standard clinical patterns. A separate investigation is planned to assess the pandemic’s impact on the epidemiology and management of hydatid disease.

## 2. Materials and Methods

The objective of this retrospective study was to conduct an in-depth analysis of patients subjected to surgical interventions for hydatid disease at the Third Surgical Clinic of Bucharest’s Emergency Clinical Hospital during a five-year interval spanning from 1 January 2015 through 31 December 2019.

### 2.1. Inclusion Criteria

The study relied on clearly defined inclusion criteria to ensure a consistent and clinically meaningful patient cohort. Only patients with a confirmed diagnosis of hydatid disease who underwent surgical treatment at our clinic during the established study period were included. A key requirement was that all patients included had received Albendazole therapy for at least four weeks prior to surgery, which was in line with recommendations from existing literature [7,17]. This protocol was routinely followed in our clinical practice, and no surgical interventions were performed without prior antiparasitic treatment.

### 2.2. Analysis of Clinical Variables and Surgical Data

Following the fulfillment of the inclusion criteria for this study, the cohort analysis encompassed a range of variables, including patient demographics such as age, gender and geographical origin, along with clinical presentations at admission, which included symptoms like abdominal or thoracic pain dyspnea palpable abdominal masses fever and jaundice. Surgical interventions were classified into two categories: total cystectomy versus partial cystectomy, with procedures being performed via both laparoscopic and open techniques. Furthermore, an assessment was made regarding recurrence rates among these patients.

Regarding the characteristics of hydatid cysts, their localization and size were analyzed, along with the presence of calcifications. Additionally, the cysts were classified according to the World Health Organization—Informal Working Group on Echinococcosis (WHO-IWGE) classification system [14]. Each patient was assigned a cyst stage (CE1–CE5) based on the imaging data available at the time of admission, primarily derived from abdominal ultrasound. These evaluations were performed during routine diagnostic workups by radiologists with experience in hepatobiliary imaging. Although our team did not include a dedicated sonographer as a named investigator, the staging was conducted retrospectively using detailed ultrasound reports documented in the medical records. The descriptions provided were sufficiently clear to allow accurate categorization in accordance with WHO guidelines.

Postoperative complications were systematically evaluated utilizing the Clavien–Dindo classification system to standardize reporting and comparison across different surgical contexts. The Clavien–Dindo classification is a grading system for postoperative complications. It ranges from Grade I (minor deviations from normal recovery) to Grade V (patient death). In parallel, the severity of bile leakage was assessed according to the standardized criteria established by the International Study Group of Liver Surgery (ISGLS), which classifies bile leaks into grades A, B, or C based on both clinical manifestations and diagnostic parameters [28]. Biliary fistulas were identified in patients exhibiting daily bile drainage exceeding 30 mL through their surgical drains that persisted beyond a duration of 48 h. These fistulas were further stratified based on output volume as either low (<300 mL/day) or high (>300 mL/day). Furthermore, an assessment of whether endoscopic interventions such as papillosphincterotomy via Endoscopic Retrograde Cholangiopancreatography (ERCP) were required added another dimension to this comprehensive evaluation assessed.

This study comprehensively analyzed several key parameters related to biliary fistulas. Specifically, the duration required for fistula closure and its mean output volume were examined in detail. Furthermore, an assessment was made regarding the length of hospitalization and the associated treatment costs for both patients who experienced uncomplicated recoveries following surgery and those who encountered postoperative complications. Lastly, a thorough evaluation of postoperative mortality rates was conducted over a 90-day period.

### 2.3. Statistical Analysis

For the statistical analysis and interpretation of results, we utilized IBM SPSS Statistics 25 software for Windows (IBM, Armonk, NY, USA). To determine if the numerical data adhered to a normal distribution, the Shapiro–Wilk test was employed with a significance level set at *p* < 0.05 to indicate normality. Descriptive statistics encompassing measures of central tendency and dispersion were applied to summarize numerical variables. For the categorical data, frequency distributions and percentages were computed to illustrate variations across different study periods. To compare the two independent groups, statistically significant differences were assessed using the Student’s *t*-test. The Mann–Whitney test was used to compare two independent groups when at least one subgroup did not follow a normal distribution. For the categorical variables, group comparisons were conducted using the Chi-square test or Fisher’s exact test, depending on the expected frequency distribution. A *p*-value less than 0.05 was deemed statistically significant across all analyses, indicating that observed findings were unlikely due to random variation alone.

## 3. Results

This retrospective study was conducted at the Third Surgical Clinic of Bucharest’s Emergency Clinical Hospital and included a cohort of 62 patients who met the inclusion criteria and underwent surgical interventions for hydatid disease treatment. Among the operated patients, 30 were males (48.33%) and 32 were females (51.61%). The mean age of the cohort was 44.1 years, with an age range spanning from 17 to 74 years. The majority of patients (*n* = 56, >90%) originated from southeastern Romania, a region endemic for hydatid disease. Regarding their place of origin, most patients came from rural areas (42 individuals, accounting for approximately 67.74%), while fewer were from urban environments (20 individuals, representing about 32.26%).

The types of symptoms and clinical signs that patients presented when they came to the hospital are presented in Table 1.

Among the 62 patients, 39 (62.9%) presented with cysts localized in the right hepatic lobe, while 23 (37.1%) had cysts situated in the left hepatic lobe. Additionally, eight patients (12.9%) exhibited intraperitoneal localization, and four patients (5.8%) had pulmonary localization. The mean size of the cysts was 10.9 ± 9.1 cm, with a range spanning from 3.5 to 40 cm. Calcifications were identified in eleven patients (17.7%). Regarding the surgical approach, 10 patients (16.12%) were treated laparoscopically, whereas 52 (83.87%) underwent traditional open surgery. The distribution of cysts according to the WHO-IWGE classification is detailed in Table 2.

Patients who underwent laparoscopic surgery had a mean cyst diameter of 10.4 ± 3.7 cm, compared to a mean diameter of 10.7 ± 5.5 cm in the open surgery group. The independent samples *t*-test revealed no statistically significant difference between the two groups (*p* = 0.620).

The type of cystectomy varied among patients. Specifically, seven patients (11.29%) underwent total cystectomy, while the majority, 55 patients (88.71%), received partial cystectomy. A total of nine patients (14.51%) presented with recurrent disease; they underwent surgical treatment for the second time for the treatment of this disease. These patients underwent surgery via a conventional approach.

### Postoperative Outcomes and Complications

Among the 62 patients, 16 (25.8%) experienced postoperative complications. The distribution of these complications according to the Clavien–Dindo classification is detailed in Table 3.

The postoperative mortality rate was 3.22% (*n* = 2 patients). The most frequent complication encountered was postoperative biliary fistula, occurring in eight patients (12.9%), which accounted for 50% of all observed complications. Additionally, three patients (4.88%) developed an abscess of the residual cavity.

Patients who developed biliary fistula had a mean age of 40.12 ± 15.94 years, compared to 44.68 ± 16.44 years in those without this complication.

The mean cyst size in patients with biliary fistula (152.13 ± 105.68 mm) was significantly larger compared to those without this complication (102.20 ± 37.86 mm), *p* = 0.012.

All patients who developed biliary fistula underwent partial cystectomy.

The distribution of hydatid cyst types according to the WHO-IWGE classification, comparing patients who developed biliary fistula with those who did not, is presented in Table 4.

According to ISGLS criteria, out of the eight patients (12.9%) who developed biliary leakage, two patients (3.2%) were classified as grade A, six patients (9.6%) as grade B, and no cases were classified as grade C.

Among these, five patients (8.06%) had a low-output fistula (according to our classification), whereas three patients (4.83%) presented with a high-output fistula. The mean fistula output volume was 437.5 ± 232.6 mL per day, ranging from a minimum of 100 mL to a maximum of 800 mL per day.

The mean fistula closure time was 23 ± 14.91 days, with a minimum of 5 days and a maximum of 50 days.

When analyzed based on fistula output, the closure duration was significantly longer in patients with high-output fistulas compared to those with low-output fistulas (29 vs. 9.3 days), *p* = 0.045.

Among patients with external biliary fistula, spontaneous closure occurred in two cases after 5 and 6 days, respectively.

For the remaining six patients, endoscopic retrograde papillosphincterotomy was required, with a mean fistula closure time of 27 days (range: 10–50 days) in these patients.

Of these patients, three (50%) subsequently developed cholangitis, which resolved under conservative treatment.

The mean hospital stay for patients with complications was significantly longer, at 26.13 ± 15.08 days, compared to those without biliary fistulas, who had a mean stay of 11.81 ± 10.18 days (*p* = 0.001). Additionally, the costs associated with hospitalization were higher for patients with biliary fistulas (€2106 ± €1694 vs. €4172 ± €1323; *p* = 0.027).

To further explore the statistical associations, we analyzed the relationship between various study variables and the presence of biliary fistula as a postoperative complication. Our analysis revealed significant correlations between the presence of biliary fistula and several factors:

- The presence of an abscess in the residual cavity (*p* = 0.004);
- A recurrence of disease (*p* = 0.03).

Additionally, a statistically significant association was found between the presence of biliary fistula and cyst size (*p* = 0.04).

## 4. Discussion

Our retrospective study included a sample of 62 patients diagnosed with hydatid disease who underwent surgical interventions at the Third Surgical Clinic of Bucharest’s Emergency Clinical Hospital. The mean age of the patients was 44.1 years, ranging from 17 to 74 years, which aligns with the literature indicating a peak incidence in the fourth and fifth decades of life [29]. The gender distribution was relatively balanced (48.33% males and 51.61% females), although previous studies suggest that women may have a predisposition to developing this pathology [30,31]. The majority of patients (>90%) originated from southeastern Romania, a region endemic to hydatid disease [32]. Regarding their place of origin, most patients came from rural areas (67.74%), highlighting the significance of socio-economic and cultural factors in disease transmission. These findings are supported by recent studies showing higher prevalence rates in rural areas due to direct contact with infected dogs and consumption of contaminated water [33,34]. The primary symptom reported by patients was abdominal or thoracic pain (79.03%), consistent with another study that noted this symptom in 67.57% of cases [35].

Among hepatic localizations, 62.9% of the cysts were identified in the right lobe and 37.1% in the left lobe. This pattern aligns with established findings in the literature, which indicate that hepatic involvement is the most common due to initial larval filtration through the portal circulation [36,37]. More than 60% of global cases present with hepatic localization, with a consistent predominance in the right lobe attributed to its vascular architecture and increased perfusion [37,38]. The mean cyst diameter recorded in this cohort was 10.9 ± 9.1 cm, closely reflecting data from similar studies, where an average size of approximately 10.6 cm was reported [35].

Surgical management in this study primarily consisted of partial cystectomy, performed in 88.71% of cases, while total cystectomy was applied in only 11.29%. This trend reflects standard surgical decision-making in scenarios where full resection may be contraindicated by anatomical constraints or increased operative risk. A laparoscopic approach was utilized in 16.12% of patients. Although less commonly employed, laparoscopy is gaining recognition for its advantages in select cases. The current literature supports its efficacy, citing faster recovery and fewer postoperative complications, with outcomes comparable to those of conventional surgery [15,39,40,41]. One study, in particular, demonstrated a significantly shorter hospitalization and lower morbidity in patients undergoing laparoscopic procedures [39]. Nonetheless, careful patient selection remains imperative, as not all cases are amenable to minimally invasive techniques without compromising safety [42].

Postoperative complications occurred in 25.8% of patients. The postoperative mortality rate was 3.22%, which is comparable to other studies reporting mortalities between 1–10% [43,44]. The most frequent complication observed was postoperative biliary fistula (12.9%), a well-documented issue in the literature [35,45]. Calcified hydatid cysts are generally considered to represent inactive or end-stage disease (CE4–CE5) [14]. These lesions are typically associated with extensive fibrosis, necrosis, and reduced intracystic pressure—factors that significantly lower the risk of rupture into the biliary tract. In contrast, active or transitional cysts (CE2–CE3) are more likely to maintain communication with the biliary system due to their higher internal pressure and ongoing parasitic activity. Consequently, the likelihood of developing postoperative biliary complications, such as biliary fistulas, appears to be lower in patients with calcified cysts [39,42].

The mean size of cysts in patients with biliary fistulas was significantly larger, at 152.13 ± 105.68 mm, compared to those without this complication, who had a mean size of 102.20 ± 37.86 mm (*p* = 0.012). These findings align with the literature indicating that larger cyst sizes increase the risk of damaging biliary structures [46,47]. Additionally, all patients with biliary fistulas underwent partial cystectomy, a technique associated with an increased risk of biliary complications due to incomplete manipulation of the cystic cavity [47].

The mean duration for fistula closure was 23 ± 14.91 days, which is significantly longer in patients with high-output fistulas (29 days) compared to those with low-output fistulas (9.3 days; *p* = 0.045). ERCP was necessary in 75% of cases of external biliary fistula to facilitate biliary drainage. This procedure proved effective in accelerating fistula closure by reducing intrahepatic pressure and promoting healing [48]. However, ERCP is not devoid of risks, as it was associated with a post-procedural cholangitis rate of 50% among patients in our cohort who developed complications. This finding aligns with recent literature reporting cholangitis rates between 30% and 60% among patients treated for external biliary fistulas via ERCP [49]. The elevated risk of post-ERCP cholangitis underscores the need for close monitoring and appropriate antibiotic prophylaxis to mitigate the risk of septic complications.

The mean hospital stay was significantly longer for patients with external biliary fistulas compared to those without complications (*p* = 0.001). Additionally, treatment costs were nearly doubled for patients with biliary fistulas (*p* = 0.027). Similar findings have been reported in other studies: one study from the western part of our country noted a hospitalization period ranging from 8 to 14 days with a mean stay of 12.6 ± 7.8 days [27], while another study with a similar cohort reported an average hospitalization of approximately 9.5 days [50]. In Italy, between 2001–2012, about half of the patients were hospitalized for less than a week, and the mean length of stay was around 12 ± 1 days [51]. In Turkey, Akkucuk et al. found an even shorter average hospitalization duration (5.42 ± 3.16 days) in liver hydatid disease cases [52].

In the present study, no significant associations were observed between preoperative laboratory findings and the incidence of postoperative biliary complications. However, it is a well-known fact that elevated levels of liver enzymes such as ALT, AST, GGT, and ALP, as well as hyperbilirubinemia, have been associated with an increased likelihood of biliary tract involvement or occult cysto-biliary communication [34,39]. These abnormalities may reflect subclinical cholestasis or early rupture into the biliary system, even in the absence of overt symptoms [46].

Statistical analysis demonstrated significant associations between external biliary fistulas and several clinical variables, including abscesses in residual cavities (*p* = 0.004), recurrence of hydatid disease (*p* = 0.03), and cyst size. Clinically, these factors are commonly encountered in patients with more complex postoperative courses. Abscess formation, recurrent disease, and larger cysts may contribute to anatomical distortion or delayed healing, thereby increasing the risk of biliary leakage. For instance, voluminous cysts can compress bile ducts or alter normal anatomy, while recurrent disease may involve fibrotic tissue planes that complicate surgical dissection [35]. Similarly, residual cavity abscesses may prolong inflammation and favor persistent leakage. These patterns have led clinicians to consider whether such variables could consistently predict postoperative complications and guide risk stratification [45]. Our findings confirm these concerns, supporting their potential use as early markers for identifying patients at increased risk of biliary fistulas and related complications.

Biliary fistulas are recognized in the literature as one of the most common post-surgical complications associated with hydatid disease interventions [50]. The size of the cyst is often correlated with the risk of their occurrence; Kilic et al. [53] reported a direct association between maximal cyst size and biliary–cyst communication, identifying 7.5 cm as a cutoff point with optimal predictive value (sensitivity 79%, specificity 73%).

Studies have shown that the presence of abscesses in residual cavities is associated with an increased incidence of biliary complications, highlighting the importance of careful postoperative evaluation of patients [43]. Furthermore, incomplete surgical interventions or inadequate management of residual cavities can significantly increase the risk of recurrence and postoperative complications [50,53]. These observations suggest that identifying predictive markers could enhance postoperative management strategies and contribute to reducing morbidity associated with surgical interventions for hydatid disease [54]. Therefore, the rigorous monitoring of patients with large hydatid cysts or abscesses is essential for preventing severe complications and optimizing clinical outcomes.

### Study Limitations

Several limitations of this study warrant consideration. Firstly, the retrospective nature of the investigation relies on the data collected from the patient’s medical records, which may not encompass all relevant epidemiological factors that could serve as potential risk factors for infection. Secondly, the database is confined to hospitalized cases in one of the three clinics of a tertiary hospital in Romania’s capital city. Consequently, there is a need for nationwide studies to analyze and interpret data comprehensively, aiming to develop protocols that significantly influence patient prognosis. Furthermore, these patients were operated on by multiple surgeons. Although the foundational principles guiding interventions remain consistent across all surgeons, individual techniques vary due to differences in experience and expertise. Despite not having an exceptionally large sample size, our findings align with numerous studies both domestically and internationally, thereby validating our results. This study highlights ongoing challenges requiring future strategic implementation for managing patients with hydatid disease effectively.

## 5. Conclusions

This study provides a comprehensive analysis of the clinical characteristics and postoperative evolution of cystic echinococcosis in patients undergoing surgical management in a tertiary care center in Romania. Biliary fistula emerged as the most prevalent postoperative complication, with a significantly higher incidence in patients presenting with larger cysts. Notably, high-output fistulas required prolonged healing, underscoring the complexity of postoperative management in these cases.

The findings highlight the critical need for a multidisciplinary approach that integrates advanced surgical techniques, endoscopic interventions, and targeted pharmacological strategies to optimize patient outcomes and minimize postoperative morbidity. Rigorous perioperative management and vigilant long-term monitoring remain paramount in enhancing clinical outcomes and improving the overall prognosis of patients with hepatic hydatid disease.

## Figures and Tables

**Table 1:** Symptoms and clinical signs.

Symptoms or Clinical Signs	Present (%)	Absent (%)
Abdominal or thoracic pain	49 (79.03%)	13 (20.96%)
Dyspnea	4 (6.45%)	58 (93.54%)
Asymptomatic	6 (9.67%)	56 (90.32%)
Palpable abdominal mass	32 (51.61%)	30 (48.39%)
Fever	9 (14.51%)	53 (85.48%)
Jaundice	6 (9.67%)	56 (90.32%)

**Table 2:** WHO-IWGE classification.

Cyst Type According to WHO-IWGE	*n* Total (%)	Open Surgery	Laparoscopic
CE 1	12 (19.35%)	9 (17.3%)	3 (30%)
CE 2	28 (45.16%)	24 (46.2%)	4 (40%)
CE 3a	6 (9.67%)	5 (9.6%)	1 (10%)
CE 3b	10 (16.12%)	9 (17.3%)	1 (10%)
CE 4	4 (6.45%)	3 (5.8%)	1 (10%)
CE 5	2 (3.22%)	2 (3.8%)	-

**Table 3:** Postoperative complications.

Clavien–Dindo Classification	
Grade I	1 (1.6%)
Grade II	7 (11.2%)
Grade IIIa	6 (9.6%)
Grade V	2 (3.2%)

**Table 4:** Comparison of cyst types. Patients with fistula vs. patients with no fistula.

Cyst Type According to WHO-IWGE	No Fistula	Fistula	*p*
CE 1	11 (17.74%)	1 (1.61%)	
CE 2	24 (38.7%)	4 (6.45%)	
CE 3a	5 (8.06%)	1 (1.61%)	0.891
CE 3b	8 (12.9%)	2 (3.22%)	
CE 4	4 (6.45%)	0	
CE 5	2 (3.22%)	0	

## Data Availability

The datasets used and/or analyzed during the current study are available from the corresponding author upon reasonable request.

## References

[1] McManus D.P., Gray D.J., Zhang W., Yang Y. (2012). Diagnosis, treatment, and management of echinococcosis. BMJ.

[2] WHO https://www.who.int/news-room/fact-sheets/detail/echinococcosis.

[3] CDC https://www.cdc.gov/echinococcosis/about/about-cystic-echinococcosis-ce.html.

[4] Ministerul Agriculturii şi Dezvoltării Rurale. https://www.madr.ro/docs/agricultura/strategia-agroalimentara-2020-2030.pdf.

[5] Moldovan R., Neghina A.M., Calma C.L., Marincu I., Neghina R. (2012). Human cystic echinococcosis in two south-western and central-western Romanian counties: A 7-year epidemiological and clinical overview. Acta Trop..

[6] Aziz H., Seda P., Aswani Y., Gosse M.D., Krishnakumari A.J., Pawlik T.M. (2025). Cystic echinococcosis of the liver. J. Gastrointest. Surg..

[7] Wen H., Vuitton L., Tuxun T., Li J., Vuitton D.A., Zhang W., McManus D.P. (2019). Echinococcosis: Advances in the 21st Century. Clin. Microbiol. Rev..

[8] Nunnari G., Pinzone M.R., Gruttadauria S., Celesia B.M., Madeddu G., Malaguarnera G., Pavone P., Cappellani A., Cacopardo B. (2012). Hepatic echinococcosis: Clinical and therapeutic aspects. World J. Gastroenterol..

[9] Thapa B., Sapkota R., Kim M., Barnett S.A., Sayami P. (2018). Surgery for parasitic lung infestations: Roles in diagnosis and treatment. J. Thorac. Dis..

[10] Badwaik N., Gharde P., Shinde R.K., Tayade H., Navandhar P.S., Patil M. (2024). Hydatid Cyst or Echinococcosis: A Comprehensive Review of Transmission, Clinical Manifestations, Diagnosis, and Multidisciplinary Treatment. Cureus.

[11] Feier CV I., Muntean C., Faur A.M., Gaborean V., Petrache I.A., Cozma G.V. (2024). Exploring Inflammatory Parameters in Lung Cancer Patients: A Retrospective Analysis. J. Pers. Med..

[12] Morar R., Feldman C. (2003). Pulmonary echinococcosis. Eur. Respir. J..

[13] Secchi M.A., Pettinari R., Mercapide C., Bracco R., Castilla C., Cassone E., Sisco P., Andriani O., Rossi L., Grondona J. (2010). Surgical management of liver hydatidosis: A multicentre series of 1412 patients. Liver Int..

[14] (2003). WHO Informal Working Group International classification of ultrasound images in cystic echinococcosis for application in clinical and field epidemiological settings. Acta Trop..

[15] Shabani M., Behnam F., Akbari H., Eidy M. (2025). Comparison of the frequency of complications resulting from open and laparoscopic surgery for hydatid cyst. Asian J. Endosc. Surg..

[16] Nasseri-Moghaddam S., Abrishami A., Taefi A., Malekzadeh R. (2011). Percutaneous needle aspiration, injection, and re-aspiration with or without benzimidazole coverage for uncomplicated hepatic hydatid cysts. Cochrane Database Syst. Rev..

[17] Horton J. (2003). Albendazole for the treatment of echinococcosis. Fundam. Clin. Pharmacol..

[18] Velasco-Tirado V., Romero-Alegría Á., Belhassen-García M., Alonso-Sardón M., Esteban-Velasco C., López-Bernús A., Carpio-Perez A., Jimenez López M.F., Muñoz Bellido J.L., Muro A. (2017). Recurrence of cystic echinococcosis in an endemic area: A retrospective study. BMC Infect. Dis..

[19] Zorbozan O. (2022). The Impact of COVID-19 Pandemic on Access to Healthcare: The Experience of the Diagnostic Parasitology Laboratory of Ege University. COVID-19 Pandemisinin Sağlık Hizmetlerine Erişime Etkisi: Ege Üniversitesi Parazitoloji Direkt Tanı Laboratuvarı Deneyimi. Turk. Parazitolojii Derg..

[20] Feier CV I., Santoro R.R., Faur A.M., Muntean C., Olariu S. (2023). Assessing Changes in Colon Cancer Care during the COVID-19 Pandemic: A Four-Year Analysis at a Romanian University Hospital. J. Clin. Med..

[21] Cozma G.V., Muntean C., Faur A.M., Gaborean V., Petrache I.A., Feier C.V.I. (2024). Unveiling the Effects of the COVID-19 Pandemic on Lung Cancer Surgery. Medicina.

[22] Feier CV I., Faur A.M., Muntean C., Blidari A., Contes O.E., Streinu D.R., Olariu S. (2023). The Challenges of Gastric Cancer Surgery during the COVID-19 Pandemic. Healthcare.

[23] Feier CV I., Muntean C., Faur A.M., Blidari A., Contes O.E., Streinu D.R., Olariu S. (2023). The Changing Landscape of Thyroid Surgery during the COVID-19 Pandemic: A Four-Year Analysis in a University Hospital in Romania. Cancers.

[24] Puia A., Feier CV I., Gaborean V., Bodea R., Graur F., Zaharie F., Al-Hajjar N., Puia I.C. (2024). Changes in Pancreatic Cancer Management and Surgical Treatment During the COVID-19 Pandemic. Medicina.

[25] Feier CV I., Bardan R., Muntean C., Feier O., Olariu A., Olariu S. (2022). The consequences of the Covid-19 pandemic on elective surgery for colon cancer. Ann. Ital. Chir..

[26] Ulusan Bağcı Ö. (2023). Evaluation of the Impact of the COVID-19 Pandemic on Cystic Echinococcosis Indirect Hemagglutination Test Dynamics: A Single-center Experience. COVID-19 Pandemisinin Kistik Ekinokokkoz İndirekt Hemaglütinasyon Test Dinamikleri Üzerindeki Etkisinin Değerlendirilmesi: Tek Merkez Deneyimi. Turk. Parazitolojii Derg..

[27] Paduraru A.A., Lupu M.A., Sima L., Cozma G.V., Olariu S.D., Chiriac S.D., Totolici B.D., Pirvu C.A., Lazar F., Nesiu A. (2023). Cystic Echinococcosis in Hospitalized Adult Patients from Western Romania: 2007–2022. Microorganisms.

[28] Koch M., Garden O.J., Padbury R., Rahbari N.N., Adam R., Capussotti L., Fan S.T., Yokoyama Y., Crawford M., Makuuchi M. (2011). Bile leakage after hepatobiliary and pancreatic surgery: A definition and grading of severity by the International Study Group of Liver Surgery. Surgery.

[29] Possenti A., Manzano-Román R., Sánchez-Ovejero C., Boufana B., La Torre G., Siles-Lucas M., Casulli A. (2016). Potential Risk Factors Associated with Human Cystic Echinococcosis: Systematic Review and Meta-analysis. PLoS Neglected Trop. Dis..

[30] Muqaddas H., Arshad M., Ahmed H., Mehmood N., Khan A., Simsek S. (2019). Retrospective Study of Cystic Echinococcosis (CE) Based on Hospital Record from Five Major Metropolitan Cities of Pakistan. Acta Parasitol..

[31] Abdulhameed M.F., Habib I., Al-Azizz S.A., Robertson I. (2018). A retrospective study of human cystic echinococcosis in Basrah province, Iraq. Acta Trop..

[32] Dărăbuș G., Bușe A., Oprescu I., Morariu S., Mederle N., Ilie M., Imre M. (2022). Investigation on Descriptive Epidemiology, Geographical Distribution, and Genotyping of *Echinococcus granulosus* s.l. in Bovine from Romania. Vet. Sci..

[33] Hajipirloo H.M., Bozorgomid A., Alinia T., Tappeh K.h.H., Mahmodlou R. (2013). Human cystic echinococcosis in west azerbaijan, northwest iran: A retrospective hospital based survey from 2000 to 2009. Iran. J. Parasitol..

[34] Al-Jawabreh A., Ereqat S., Dumaidi K., Nasereddin A., Al-Jawabreh H., Azmi K., Al-Laham N., Nairat M., Casulli A., Maqboul H. (2017). The clinical burden of human cystic echinococcosis in Palestine, 2010–2015. PLoS Neglected Trop. Dis..

[35] Gökçe O.N., Alkan S., Karadağ V. (2024). Cystic Echinococcosis Cases: A Retrospective Evaluation in a General Surgery Department. Infect. Dis. Clin. Microbiol..

[36] van Cauteren D., Millon L., de Valk H., Grenouillet F. (2016). Retrospective study of human cystic echinococcosis over the past decade in France, using a nationwide hospital medical information database. Parasitol. Res..

[37] Colombe S., Togami E., Gelaw F., Antillon M., Fuentes R., Weinberger D.M. (2017). Trends and correlates of cystic echinococcosis in Chile: 2001–2012. PLoS Neglected Trop. Dis..

[38] Bhutani N., Kajal P. (2018). Hepatic echinococcosis: A review. Ann. Med. Surg..

[39] Bayrak M., Altıntas Y. (2019). Current approaches in the surgical treatment of liver hydatid disease: Single center experience. BMC Surg..

[40] Masood P.F., Mufti G.N., Wani S.A., Sheikh K., Baba A.A., Bhat N.A., Hamid R. (2022). Comparison of laparoscopic and open surgery in hepatic hydatid disease in children: Feasibility, efficacy and safety. J. Minimal Access Surg..

[41] Shaikh O., Kumbhar U., Bhattarai S., Chilaka S., Reddy N., Tajudeen M. (2021). Feasibility of Laparoscopic Closed Cystectomy for Hepatic Hydatid Cyst in Segments VI, VII, and VIII. Cureus.

[42] Tai Q.W., Tuxun T., Zhang J.H., Zhao J.M., Cao J., Muhetajiang M., Bai L., Cao X.L., Zhou C.M., Ji X.W. (2013). The role of laparoscopy in the management of liver hydatid cyst: A single-center experience and world review of the literature. Surg. Laparosc. Endosc. Percutaneous Tech..

[43] Daradkeh S., El-Muhtaseb H., Farah G., Sroujieh A.S., Abu-Khalaf M. (2007). Predictors of morbidity and mortality in the surgical management of hydatid cyst of the liver. Langenbeck’s Arch. Surg..

[44] Shi H., Tuerxun K., Yusupu A., Yusupu Z., Xu Q., Jia Y., Maimaitireyimu M., Maimaitiaili T., Muhetajiang M., Lin J. (2024). Perioperative outcomes and hospitalization costs of radical vs. conservative surgery for hepatic cystic echinococcosis: A retrospective study. PLoS Neglected Trop. Dis..

[45] Smego R.A., Sebanego P. (2005). Treatment options for hepatic cystic echinococcosis. Int. J. Infect. Dis. IJID.

[46] Jerraya H., Khalfallah M., Osman S.B., Nouira R., Dziri C. (2015). Predictive factors of recurrence after surgical treatment for liver hydatid cyst. Surg. Endosc..

[47] Prousalidis J., Kosmidis C., Anthimidis G., Kapoutzis K., Karamanlis E., Fachantidis E. (2012). Postoperative recurrence of cystic hydatidosis. Can. J. Surg..

[48] El-Gendi A.M., El-Shafei M., Bedewy E. (2018). The Role of Prophylactic Endoscopic Sphincterotomy for Prevention of Postoperative Bile Leak in Hydatid Liver Disease: A Randomized Controlled Study. J. Laparoendosc. Adv. Surg. Tech. Part A.

[49] Bishay K., Meng Z.W., Khan R., Gupta M., Ruan Y., Vaska M., Iannuzzi J., O’Sullivan D.E., Mah B., Partridge AC R. (2025). Adverse Events Associated with Endoscopic Retrograde Cholangiopancreatography: Systematic Review and Meta-Analysis. Gastroenterology.

[50] Martel G., Ismail S., Bégin A., Vandenbroucke-Menu F., Lapointe R. (2014). Surgical management of symptomatic hydatid liver disease: Experience from a Western centre. Can. J. Surg..

[51] Brundu D., Piseddu T., Stegel G., Masu G., Ledda S., Masala G. (2014). Retrospective study of human cystic echinococcosis in Italy based on the analysis of hospital discharge records between 2001 and 2012. Acta Trop..

[52] Akkucuk S., Aydogan A., Ugur M., Yetim I., Davran R., Oruc C., Kilic E., Temiz M. (2014). Comparison of surgical procedures and percutaneous drainage in the treatment of liver hydatide cysts: A retrospective study in an endemic area. Int. J. Clin. Exp. Med..

[53] Kilic M., Yoldas O., Koc M., Keskek M., Karakose N., Ertan T., Gocmen E., Tez M. (2008). Can biliary-cyst communication be predicted before surgery for hepatic hydatid disease: Does size matter?. Am. J. Surg..

[54] Shafiei R., Mohajerzadeh M.S., Masomi H.F.A., Tavakoli M., Turki H., Firouzeh N. (2024). Discordance Therapeutic Protocol of Cystic Echinococcosis With WHO Guideline: A Descriptive Study Based on Liver Ultra-Sonographic Data in North Khorasan Province, Northeastern of Iran. J. Ultrasound Med..

